# Tofacitinib Mitigates the Increased SARS-CoV-2 Infection Susceptibility Caused by an IBD Risk Variant in the PTPN2 Gene

**DOI:** 10.1016/j.jcmgh.2024.101447

**Published:** 2025-01-03

**Authors:** Marianne R. Spalinger, Golshid Sanati, Pritha Chatterjee, Rong Hai, Jiang Li, Alina N. Santos, Tara M. Nordgren, Michel L. Tremblay, Lars Eckmann, Elaine Hanson, Michael Scharl, Xiwei Wu, Brigid S. Boland, Declan F. McCole

**Affiliations:** 1 Division of Biomedical Sciences, School of Medicine, University of California Riverside, Riverside, California; 2 Department of Gastroenterology and Hepatology, University Hospital Zurich, and University of Zurich, Zurich, Switzerland; 3 Department of Microbiology and Plant Pathology, University of California Riverside, Riverside, California; 4 Department of Biochemistry and Goodman Cancer Research Centre, McGill University, Montreal, Quebec, Canada; 5 Division of Gastroenterology, University of California San Diego, La Jolla, California; 6 Integrative Genomics Core, Beckman Research Institute of City of Hope, Monrovia, California; 7 Current position: College of Veterinary Medicine, Colorado State University, Fort Collins, Colorado

**Keywords:** Autoimmune Disease, Coronavirus, COVID-19, SARS-CoV-2, Genetic Susceptibility, Inflammatory Bowel Disease, Tofacitinib

## Abstract

**Background & Aims:**

Coronavirus disease (COVID-19), caused by severe acquired respiratory syndrome-Coronavirus-2 (SARS-CoV-2), triggered a global pandemic with severe medical and socioeconomic consequences. Although fatality rates are higher among the elderly and those with underlying comorbidities, host factors that promote susceptibility to SARS-CoV-2 infection and severe disease are poorly understood. Although individuals with certain autoimmune/inflammatory disorders show increased susceptibility to viral infections, there is incomplete knowledge of SARS-CoV-2 susceptibility in these diseases. The aim of our study was to investigate whether the autoimmunity risk gene, *PTPN2,* which also confers elevated risk to develop inflammatory bowel disease, affects susceptibility to SARS-CoV-2 viral uptake.

**Methods:**

Using samples from *PTPN2* genotyped patients with inflammatory bowel disease, *PTPN2-*deficient mice, and human intestinal and lung epithelial cell lines, we investigated how *PTPN2* affects expression of the SARS-CoV-2 receptor angiotensin converting enzyme 2 (ACE2), and uptake of virus-like particles expressing the SARS-CoV2 spike protein and live SARS-CoV-2 virus.

**Results:**

We report that the autoimmune *PTPN2* loss-of-function risk variant rs1893217 promotes expression of the SARS-CoV-2 receptor, ACE2, and increases cellular entry of SARS-CoV-2 spike protein and live virus. Elevated ACE2 expression and viral entry were mediated by increased Janus kinase-signal transducers and activators of transcription signaling and were reversed by the Janus kinase inhibitor, tofacitinib.

**Conclusion:**

Collectively, our findings uncover a novel risk biomarker for increased expression of the SARS-CoV-2 receptor and viral entry, and identify a clinically approved therapeutic agent to mitigate this risk.

## Graphical abstract

**Figure:**
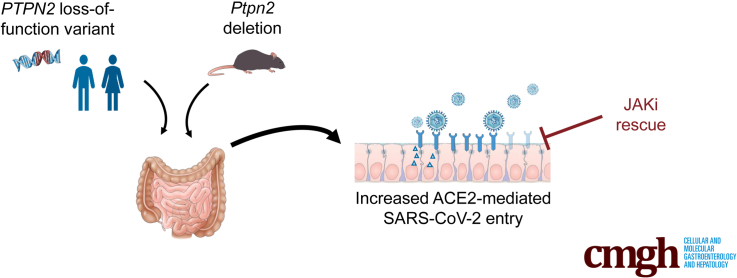

**Summary**

We identified that protein tyrosine phosphatase non-receptor type 2 restricts the expression of the severe acquired respiratory syndrome-Coronavirus-2 receptor angiotensin converting enzyme 2, resulting in elevated uptake of severe acquired respiratory syndrome-Coronavirus-2 viral particles and live virus. Elevated uptake of viral particles was normalized upon treatment with the clinically approved Janus kinase inhibitor Tofacitinib.

Since the start of the coronavirus disease (COVID-19) outbreak in late 2019, COVID-19 emerged as a severe global health, economic, and social crisis.^1^ As per January 12, 2025, COVID-19 affected almost 780 million patients, resulting in 7.1 million deaths (World Health Organization COVID-19 Dashboard; https://data.who.int/dashboards/covid19/cases). Although the initial pandemic has ceased, COVID-19 cases continue to rise and are still associated with long-term sequelae and significant morbidity.^2^ Despite tremendous effort to understand COVID-19 pathogenesis, genetic risk factors for severe disease and factors that promote the development of long COVID are still poorly defined. Although most attention has focused on symptoms in the airways, gastrointestinal (GI) symptoms were reported in 46% of all cases and 33% presented with GI symptoms in the absence of respiratory symptoms.^3^^,^^4^ GI symptoms are associated with longer duration and more severe COVID-19 (eg, increased prevalence of acute renal insufficiency^5^), emphasizing their importance for early diagnosis and prognosis.^6^ Of importance, severe acquired respiratory syndrome-Coronavirus-2 (SARS-CoV-2) can directly infect intestinal epithelial cells,^7^^,^^8^ and studies in human enteroids identified that SARS-CoV-2 can directly stimulate Ca^2+^-driven chloride secretion—a possible mechanism contributing to COVID-19 diarrhea—and inflammatory cytokine secretion.^9^ Moreover, viral particles have been detected in feces even after virus clearance from the respiratory tract.^10^^,^^11^ This indicates viral shedding in the gut, which may serve as a reservoir of virus replication, and possible oral-fecal transmission although presence of live virus in feces is disputed.^7^^,^^12^

SARS-CoV-2 entry into host cells is mediated by its spike glycoprotein (S protein), which is cleaved by cell surface-associated transmembrane protease serine protease 2 (TMPRSS2) and TMPRSS4 to generate the S1 and S2 subunits in a so-called ‘priming’ process.^12^^,^^13^ S1 binds to angiotensin I converting enzyme 2 (ACE2), a monocarboxypeptidase controlling cleavage of several peptides within the renin-angiotensin system.^12^, ^13^, ^14^ S2 drives the subsequent fusion of viral and host membranes.^15^ Interferon (IFN)-Janus kinase (JAK)- signal transducers and activators of transcription (STAT) signaling is a suggested major driver of ACE2 expression likely via STAT1/3 binding sites in the ACE2 promoter.^16^ ACE2, TMPRSS2, and TMPRSS4 are highly expressed on the surface of epithelial cells such as lung type 2 pneumocytes and absorptive intestinal epithelial cells.^7^^,^^15^, ^16^, ^17^, ^18^

About 16% to 20% of the general population carries the single nucleotide polymorphism (SNP) rs1893217 located in the gene locus encoding protein tyrosine phosphatase non-receptor type 2 (PTPN2, also called TCPTP).^19^^,^^20^ This SNP causes PTPN2 loss of function and is associated with increased risk for chronic inflammatory and autoimmune diseases including inflammatory bowel disease (IBD), type 1 diabetes (T1D), and rheumatoid arthritis (RA).^20^^,^^21^ Of special importance regarding gastrointestinal symptoms and gastrointestinal (viral) infections is the role of PTPN2 in intestinal epithelial cell (IEC) barrier protection, especially in mitigating the effects of inflammatory cytokines.^22^^,^^23^ PTPN2 directly dephosphorylates several transducers of cytokine receptor signaling including the STAT family of transcription factors (STATs 1/3/5/6) and JAK1 and JAK3 that are activated by inflammatory cytokines such as IFNγ.^22^, ^23^, ^24^ JAK inhibitors have emerged as an effective new therapeutic class in many patients with autoimmune diseases. Indeed a JAK-inhibitor, baricitinib, was shown in the ACTT-2 clinical trial to reduce disease severity and hospitalization time in patients with COVID-19 receiving the antiviral drug remdesevir. Tofacitinib (Xeljanz), a pan-JAK inhibitor that preferentially inhibits JAK1 and JAK3, is approved to treat RA and the IBD subtype ulcerative colitis (UC). We have shown that tofacitinib corrects the barrier-disrupting consequences upon PTPN2-loss in IECs or macrophages in mice *in vivo*.^25^

Using intestinal samples and peripheral blood mononuclear cells (PBMCs) from patients with IBD harboring the autoimmune *PTPN2* risk allele in SNP rs1893217, IEC lines modified by CRISPR-Cas9 to express the *PTPN2* rs1893217 variant, PTPN2-knockdown (KD) human intestinal and lung epithelial cell lines as well as *Ptpn2-*deficient mouse models, we determined that loss of PTPN2 activity promotes ACE2 expression and increased entry of viral particles expressing SARS-CoV-2 spikes. Elevated ACE2 expression and viral entry were mediated by increased epithelial JAK-STAT signalling, and were reversed by the clinically approved JAK inhibitor, tofacitinib. Collectively, our findings describe a risk factor for increased SARS-CoV-2 invasion (entry) and identify a clinically approved drug that may be utilized to mitigate this risk.^26^

## Results

### PTPN2 Regulates ACE2 Expression in vivo and in vitro

**Figure 1:**
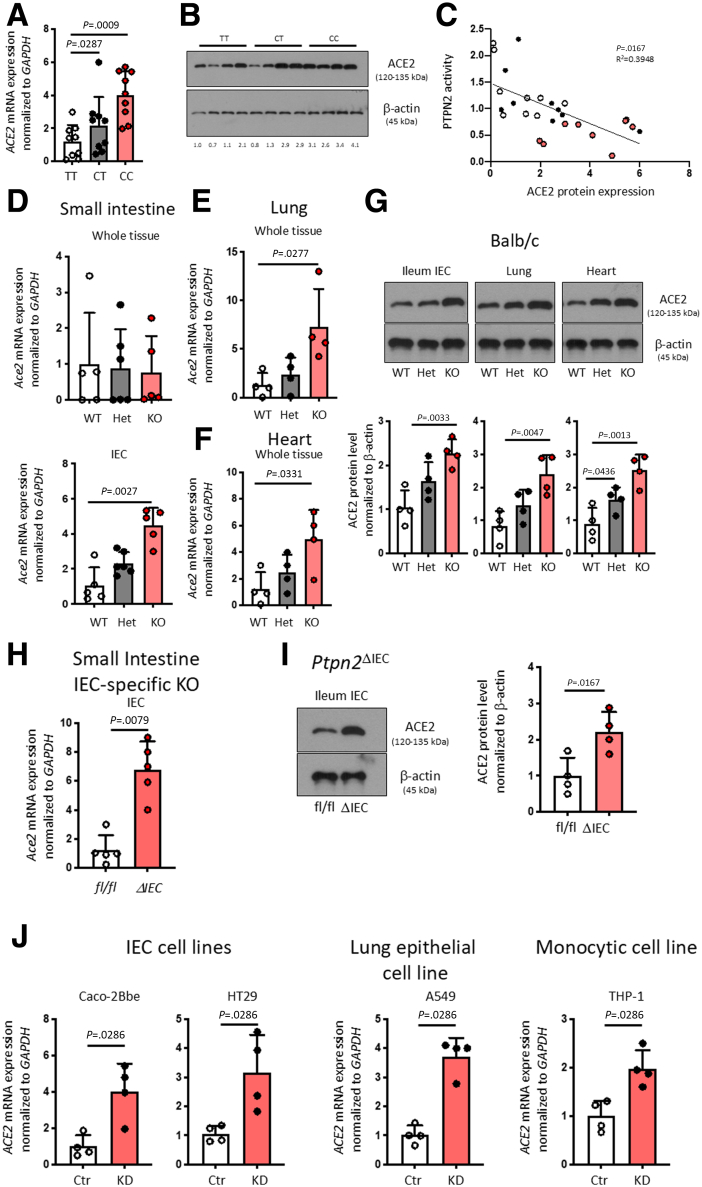
**Presence of SNP rs1893217 in *PTPN2* promotes ACE2 expression.** (*A*) Ileum and colon biopsies from patients with IBD homozygous for the major allele (TT), heterozygous (CT) or homozygous for the inflammatory disease-associated minor allele (CC) in *PTPN2* SNP rs1893217 were analyzed for *ACE2* mRNA (*A*) and ACE2 protein (*B*) expression. Depicted are representative Western blot pictures and values below the blot indicate relative band density normalized to β-actin and TT controls. (*C*) PTPN2 phosphatase activity levels were analyzed in the same samples as in *B* and correlated with relative ACE2 protein levels. (*D*) *Ace2* mRNA (normalized to *Gapdh*) in whole intestinal tissue, isolated IECs, (*E*) lung tissue, and (*F*) heart tissue from 3-week-old WT, whole-body *Ptpn2* Het or *Ptpn2* KO mice. (*G*) Representative Western blot pictures and respective densitometric analysis for ACE2 protein and β-actin in IECs from the ileum, whole lung tissue, and whole heart tissue from *Ptpn2*-WT, *Ptpn2*-Het, or *Ptpn2*-KO mice. (*H*) *Ace2* mRNA (normalized to *Gapdh*) and (*I*) ACE2 Western blot and protein densitometric analysis of small intestinal IECs isolated from mice in which *Ptpn2* was specifically deleted in IECs (ΔIEC), or their control littermates (fl/fl). Data are normalized to *Gapdh* and the average of WT or fl/fl mice, respectively. (*J*) Caco-2BBe, HT-29.cl19A, A549, and THP-1 cells expressing non-targeting control (Ctr) or *PTPN2-*specific (KD) shRNA were analyzed for mRNA expression of *ACE2.* Data are normalized to *GAPDH* and the average of Ctr shRNA expressing cells. Statistical differences are indicated in the figures (1-way ANOVA [*A+B, D+E*] or linear regression [*C*]), *A–C*, n = 8 per genotype; *E–J*, n = 4-6. Each dot represents a biological replicate.

**Figure 2:**
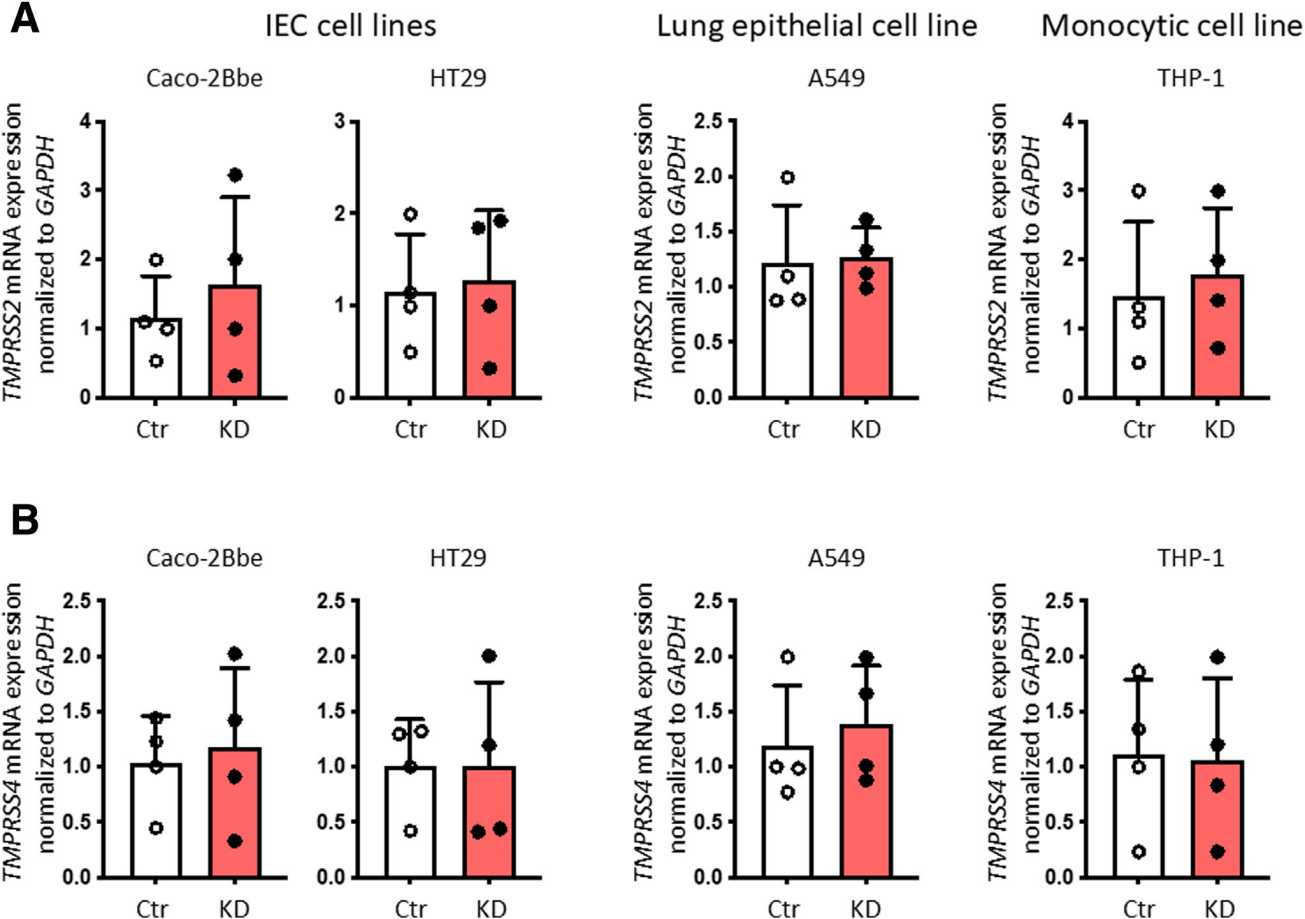
**Loss of PTPN2 did not affect expression of TMPRSS2 and TMPRSS4.** Caco-2BBe, HT-29.cl19A, A549, and THP-1 cells expressing non-targeting control (Ctr) or *PTPN2-*specific (KD) shRNA were analyzed for mRNA expression of (*A*) *TMPRSS2* and (*B*) *TMPRSS4.* Data are normalized to *GAPDH* and the average of Ctr-shRNA-expressing cells. Statistical differences are indicated in the figures (Student *t*-test; n = 4). Each dot represents the average of an independent experiment with 2 to 3 technical replicas.

Mucosal biopsy samples from patients with IBD in the Swiss IBD cohort previously genotyped for the IBD-associated loss-of-function SNP *rs1893217* in *PTPN2*^27^ were subjected to RNA sequencing. Gene ontogeny (GO) biological pathway analysis using the Database for Annotation, Visualization and Integrated Discovery (DAVID) indicated “***Digestion***” as the most significantly regulated function based on *PTPN2* genotype (presence of risk ‘C’ allele, patients with the CT or the CC genotype) independent of disease severity (Table 1). This biological process includes the *ACE2* gene, which was also found in 3 other processes that were increased in ‘C’ allele carriers (Table 1). Quantitative polymerase chain reaction (PCR) and Western blotting on intestinal biopsies isolated from patients with Crohn’s disease (CD) and UC (Table 2) confirmed increased mRNA and protein expression of ACE2 in C-allele carriers (Figure 1*A and B*). Furthermore, ACE2 protein expression negatively correlated with PTPN2 phosphatase activity (Figure 1*C*). To confirm these findings in *Ptpn2-*knockout (KO) mice, which exhibit a severe inflammatory phenotype and die within few weeks after birth,^28^ we explored ACE2 expression in 3-week-old mice when the intestinal epithelium appears relatively normal compared with heterozygous (Het) and wild-type (WT) littermates. Although *Ptpn2*-KO mice did not exhibit any difference in *Ace2* mRNA expression in whole intestinal samples compared with WT mice, *Ace2* mRNA expression in IECs from these mice was significantly increased (Figure 1*D*). In addition, *Ace2* mRNA expression in lung and cardiac tissue was significantly increased in *Ptpn2*-KO mice (Figure 1*E, F, H*), whereas ACE2 protein was increased in IECs and lung and heart tissue in *Ptpn2*-KO mice (Figure 1*G*). Given the strong increase of ACE2 expression in IECs of *Ptpn2*-KO mice, and to explore whether PTPN2 regulates ACE2 in the absence of inflammation, we confirmed these findings in mice lacking PTPN2 specifically in IECs (*Ptpn2*^ΔIEC^ mice). Also in these mice, *Ace2* mRNA and protein expression were clearly elevated in PTPN2-deficient IECs (Figure 1*H, I*), demonstrating that the increase in *Ace2* expression was not dependent on inflammation. This effect was confirmed in intestinal epithelial, lung epithelial, and monocyte cell lines upon *PTPN2* KD, where depletion of *PTPN2* resulted in elevated *ACE2* expression (Figure 1*J*).^23^ Notably, the serine proteases TMPRSS2 and TMPRSS4, which are additional cofactors of SARS-CoV-2 viral entry, were not altered in PTPN2-KD cells (Figure 2). This suggests that PTPN2 specifically regulates *ACE2* expression.

**Table 1:** Biological Processes Upregulated in Patients With IBD With the *PTPN2* rs1893217 Variant

Category	ID	Term	%	*P*-value	Genes	Benjamini
GOTERM_BP_FAT	GO:0007586	Digestion	6.67	1.21E−04	AKR1C2, MUC3A, SLC5A1, **ACE2**, PRSS1, FABP2	7.40E−02
GOTERM_BP_FAT	GO:0022600	Digestive system process	3.33	1.40E−02	MUC3A, SLC5A1, FABP2	9.89E−01
GOTERM_BP_FAT	GO:0055114	Oxidation reduction	10	1.98E−02	AKR1C3, ALDH1A1, AKR7L, AKR1C2, CYP3A5, CYP2C19, CYP2C18, CYP2C8, CYBRD1	9.86E−01
GOTERM_BP_FAT	GO:0055085	Transmembrane transport	8.89	3.13E−02	SLC5A1, KCNH6, SLC5A9, MFSD2A, ABCC2, SLC46A3, ABCC8, FLVCR1	9.94E−01
GOTERM_BP_FAT	GO:0001991	Regulation of systemic arterial blood pressure by circulatory renin-angiotensin	2.22	4.18E−02	**ACE2**, PCSK5	9.96E−01
GOTERM_BP_FAT	GO:0010817	Regulation of hormone levels	4.44	4.65E−02	SHBG, **ACE2**, PCSK5, SMPD3	9.94E−01
GOTERM_BP_FAT	GO:0003081	Regulation of systemic arterial blood pressure by renin-angiotensin	2.22	5.70E−02	**ACE2**, PCSK5	.995182193
GOTERM_BP_FAT	GO:0050892	Intestinal absorption	2.22	8.19E−02	SLC5A1, FABP2	.998877071
GOTERM_BP_FAT	GO:0043043	Peptide biosynthetic process	2.22	8.68E−02	GGT2, PCSK5	.998362738

NOTE: ACE2 highlighted in Bold.

**Table 2:** Characteristics of *PTPN2* rs1893217 Variant Genotyped Patients With IBD (CD or UC)

Genotype	Disease	Severity	Location	Inflamed	Gender (M/F)	Type of sample
TT	UC	Quiescent	Ileum	No	M	RNA
TT	CD	Moderate	Ileum	Yes	M	RNA
TT	CD	Quiescent	Rectum	No	M	RNA
TT	UC	Moderate	Rectum	Yes	M	RNA
TT	UC	Moderate	Ileum	Yes	F	RNA, Protein, PBMC
TT	UC	Quiescent	Ileum	No	F	RNA, Protein, PBMC
TT	CD	Severe	Rectum	Yes	F	RNA, Protein, PBMC
TT	CD	Moderate	Rectum	Yes	M	RNA, Protein, PBMC
TT	CD	Quiescent	Rectum	No	M	RNA, Protein, PBMC
TT	UC	Moderate	Ileum	Yes	M	Protein, PBMC
TT	UC	Quiescent	Ileum	No	M	Protein
TT	UC	Severe	Rectum	Yes	F	Protein
TT	CD	Quiescent	Ileum	No	F	Protein
CT	UC	Quiescent	Ileum	No	M	RNA
CT	CD	Moderate	Ileum	Yes	M	RNA
CT	CD	Quiescent	Rectum	No	M	RNA
CT	UC	Moderate	Rectum	Yes	M	RNA
CT	UC	Moderate	Ileum	Yes	F	RNA, Protein, PBMC
CT	UC	Quiescent	Ileum	No	F	RNA, Protein, PBMC
CT	CD	Severe	Rectum	Yes	F	RNA, Protein, PBMC
CT	CD	Moderate	Rectum	Yes	M	RNA, Protein, PBMC
CT	CD	Quiescent	Rectum	No	M	RNA, Protein, PBMC
CT	UC	Moderate	Ileum	Yes	M	Protein, PBMC
CT	UC	Quiescent	Ileum	No	M	Protein
CT	UC	Severe	Rectum	Yes	F	Protein
CT	CD	Quiescent	Ileum	No	F	Protein
CC	UC	Quiescent	Ileum	No	M	RNA
CC	CD	Severe	Ileum	Yes	M	RNA
CC	CD	Quiescent	Rectum	No	M	RNA
CC	UC	Moderate	Rectum	Yes	M	RNA
CC	UC	Moderate	Ileum	Yes	F	RNA + Protein
CC	UC	Quiescent	Ileum	No	F	RNA + Protein
CC	CD	Severe	Rectum	Yes	F	RNA + Protein
CC	CD	Moderate	Rectum	Yes	M	RNA + Protein
CC	CD	Quiescent	Rectum	No	M	RNA + Protein
CC	UC	Moderate	Ileum	Yes	M	Protein
CC	UC	Quiescent	Ileum	No	M	Protein
CC	UC	Severe	Rectum	Yes	F	Protein
CC	CD	Quiescent	Ileum	No	F	Protein

CD, Crohn’s disease; F, female; IBD, inflammatory bowel disease; M, male; PBMC, peripheral blood mononuclear cell; UC, ulcerative colitis.

### PTPN2 Negatively Regulates SARS-CoV-2 Spike Protein-mediated Viral Entry Into Epithelial and Immune Cells

**Figure 3:**
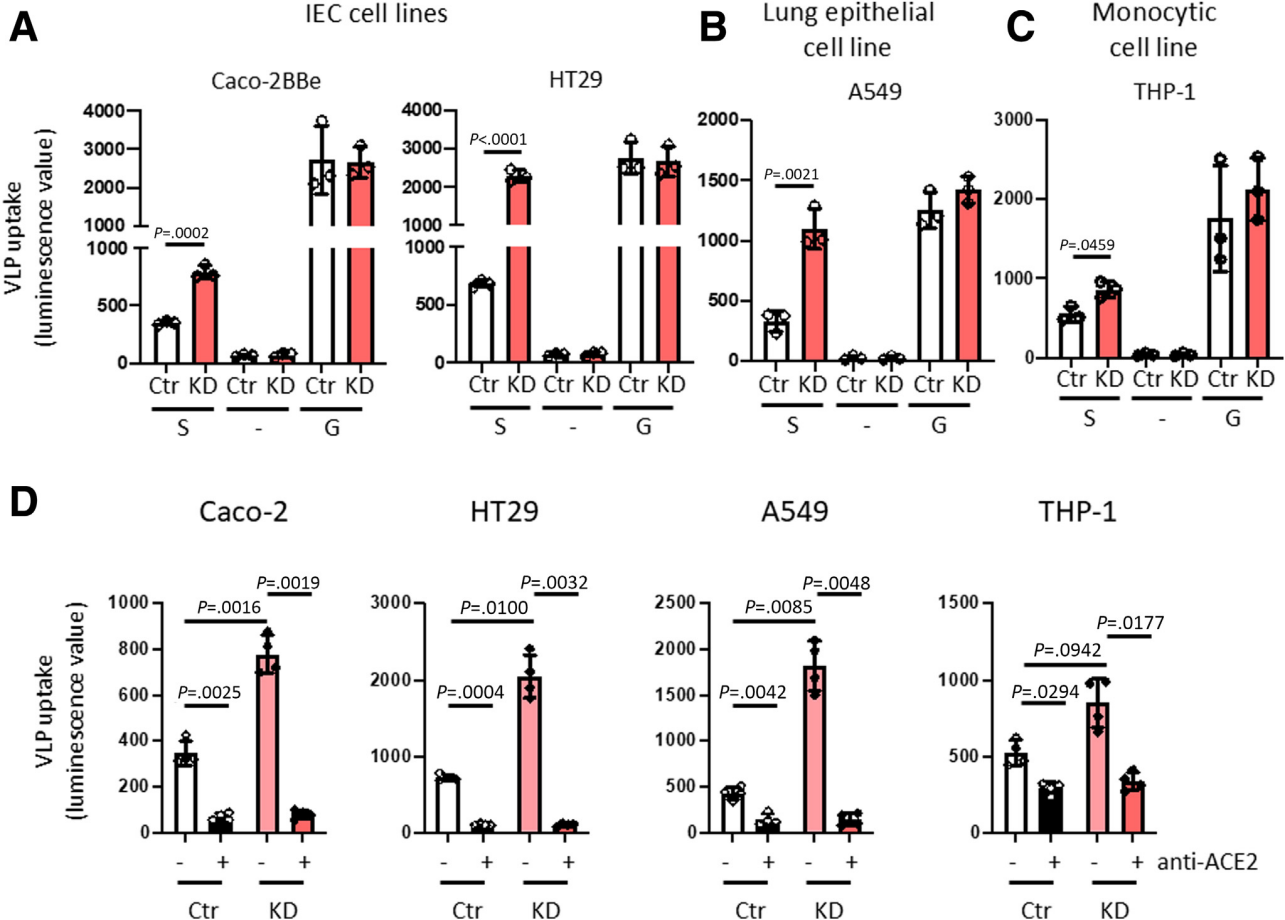
**PTPN2 knockdown facilitated ACE2-mediated entry of VLPs expressing SARS-CoV-2 spike S protein.** (*A*) Caco-2BBe; (*B*) HT-29.cl19A; (*C*) A549; and (*D*) THP-1 cells expressing non-targeting control (Ctr) or *PTPN2-*specific (KD) shRNA were incubated with VLPs expressing renilla luciferase and SARS-CoV-2 spike protein (S), no additional proteins (−, negative control), or the spike G glycoprotein of the rhabdovirus vesicular stomatitis virus (G, positive control). Forty-eight hours after infection, luminescence values of the supernatant were measured as an approximation of VLP uptake. (*D*) Caco-2BBe, HT-29.cl19A, A549, and THP-1 cells expressing non-targeting control (Ctr) or *PTPN2-*specific (KD) shRNA were incubated with an inhibitory anti-ACE2 antibody prior to infection (48 hours) with VLPs expressing SARS-CoV-2 spike protein. Statistical differences are indicated in the figure (Student *t*-test, n = 3 independent experiments; 1-way ANOVA, n = 4). Each dot represents the average of an independent experiment with 2 to 3 technical replicas each.

To assess the functional consequence of increased ACE2 expression in PTPN2-deficient cells, we assessed whether deletion of PTPN2 affects the uptake of virus-like particles (VLPs) expressing SARS-CoV-2 spikes. Apical uptake of empty VLPs (-), which served as a negative control, or uptake of VLPs covered with spike G glycoprotein of the rhabdovirus vesicular stomatitis virus (G), which served as a positive control for viral entry, was not affected in PTPN2-KD Caco-2BBe (Figure 3*A*), HT-29.cl19A IECs (Figure 3*B*), or A549 lung epithelial cells (Figure 3*C*), indicating that non-specific uptake was not affected upon PTPN2 knockdown. In contrast, VLPs expressing SARS CoV-2 spikes entered PTPN2-KD epithelial cells more efficiently than they entered control cells (Figure 2*A, B*), indicating that PTPN2 deficiency promotes ACE2 expression and viral uptake into intestinal and lung epithelial cells. Notably, loss of PTPN2 also caused a significant increase in ACE2 expression in PTPN2-KD monocytes (Figure 1*J*) and increased CoVS entry (Figure 3*C*). This indicates that PTPN2 regulation of ACE2 and SARS-CoV-2 spike-expressing VLP entry is not restricted to epithelial cells but has similar functional consequences in immune cells. Furthermore, increased uptake of SARS-CoV-2 spike-expressing VLPs in PTPN2-deficient cells was no longer observed upon inhibition of ACE2 with a blocking antibody (Figure 3*D*), indicating that ACE2 mediated the uptake of SARS-CoV-2 spike-expressing VLPs. In summary, this indicates that loss of PTPN2 promotes SARS-CoV-2 uptake by promoting ACE2 expression.

### IFN-γ Promotes ACE2 Expression and SARS-CoV-2 Spike Protein Uptake

**Figure 4:**
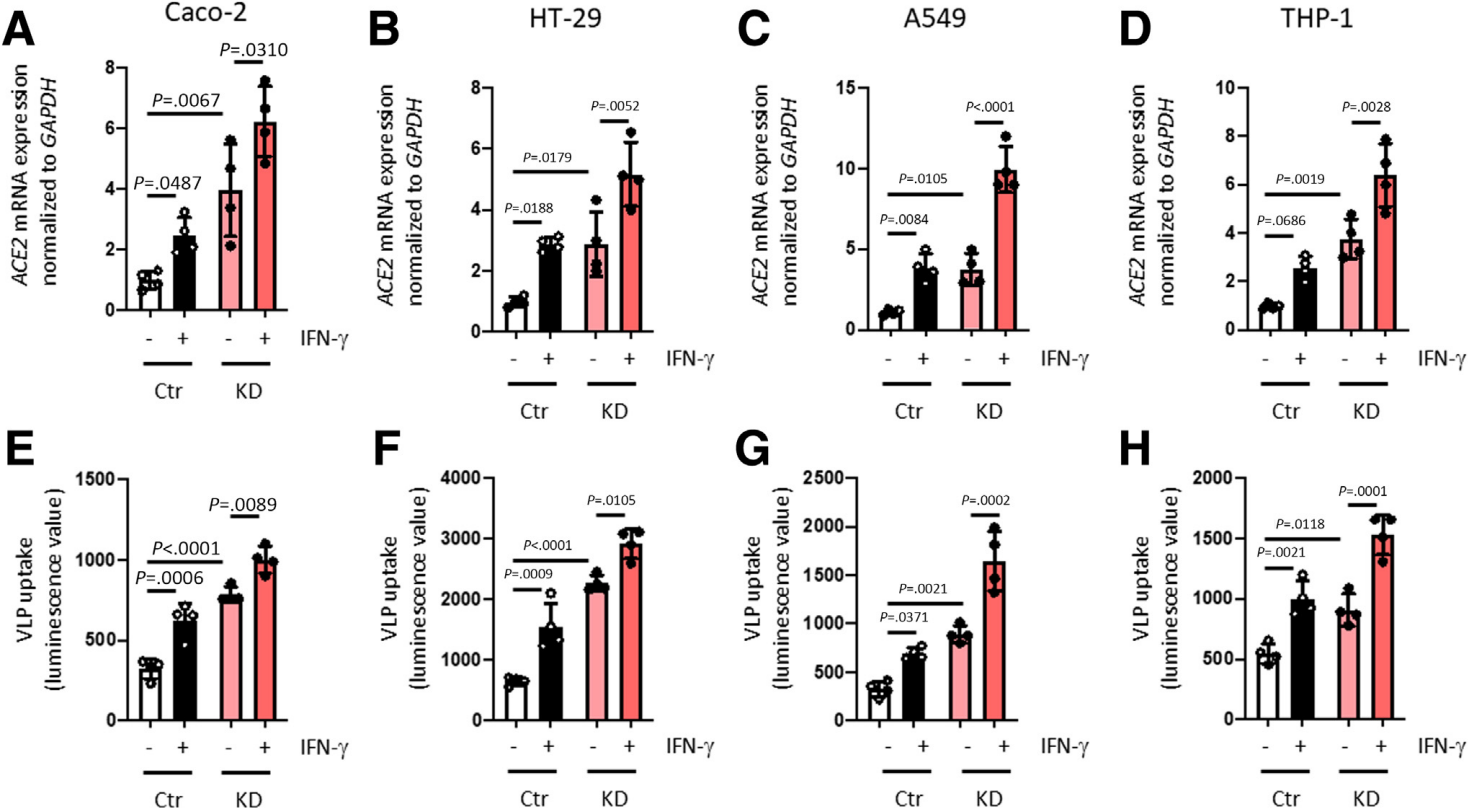
**IFN-γ promoted ACE2 expression and uptake of SARS-CoV-2 spike-expressing VLPs in intestinal and lung epithelial cells, and in monocytes.***(A–D)* Caco-2BBe, HT-29.cl19A, A549, and THP-1 cells expressing non-targeting control (Ctr) or *PTPN2-*specific (KD) shRNA were treated with IFN-γ (1000 IU/mL) for 24 hours and analyzed for *ACE2* mRNA expression, normalized to *GAPDH*. (*E–H*) Ctr and KD Caco-2BBe, HT-29.cl19A, A549, and THP-1 cells were infected with VLPs expressing SARS-CoV-2 spike protein in the presence or absence of IFN-γ (1000 IU/mL), and luminescence was measured after 48 hours. Statistical differences are indicated in the figure (1-way ANOVA, n = 4). Each dot represents the average of an independent experiment with 2 to 3 technical replicas.

**Figure 5:**
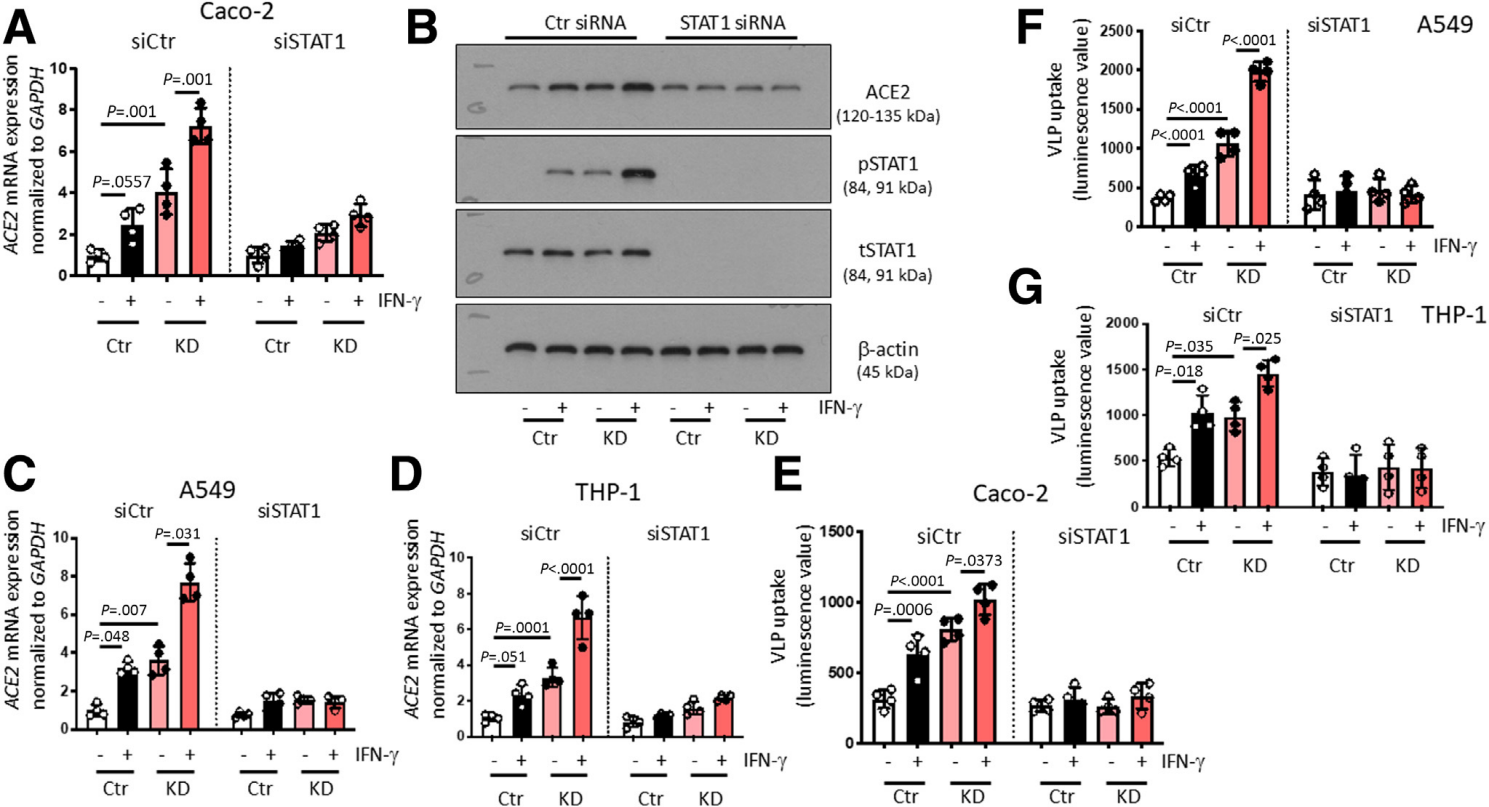
**IFN-γ promoted uptake of SARS-CoV-2 spike-expressing VLPs in a STAT1-dependent manner.** (*A*) Caco-2BBe cells expressing non-targeting control (Ctr) or *PTPN2-*specific (KD) shRNA were treated with non-targeting control (siCtr) or STAT1-specific (siSTAT1) siRNA prior to incubation with IFN-γ (1000 IU/mL) for 24 hours and analysis for *ACE2* mRNA expression. (*B*) Representative Western blot images for the indicated proteins from cells treated as in *A*. (*C, D*) Ctr and PTPN2-KD A549 lung epithelial cells and THP-1 monocytes were treated with non-targeting control (siCtr) or STAT1-specific (siSTAT1) siRNA prior to incubation with IFN-γ (1000 IU/mL) for 24 hours and analysis for *ACE2* mRNA expression. (*E–G*) Ctr and KD Caco-2BBe, A549, or THP-1 cells were treated with non-targeting control (siCtr) or STAT1-specific (siSTAT1) siRNA prior to infection with VLPs expressing SARS-CoV-2 spike protein in the presence or absence of IFN-γ (1000 IU/mL), and luminescence was measured after 48 hours. Statistical differences are indicated in the figure (1-way ANOVA, n = 4). Each dot represents the average of an independent experiment with 2 to 3 technical replicas each.

It has been suggested that ACE2 expression is induced by interferons,^29^ and its promoter is reported to have putative STAT1 binding sites,^16^^,^^30^ although newer findings debate whether interferons can induce ACE2 expression, or if it drives expression and release of a shorter version of the protein.^31^ Because PTPN2 is a potent suppressor of IFN-γ-induced signaling cascades,^22^^,^^32^ and directly dephosphorylates STAT1,^33^ we assessed whether IFN-γ treatment promotes ACE2 expression in our PTPN2-deficient cell culture models. Indeed, IFN-γ promoted ACE2 expression, and this was further increased in PTPN2-KD IECs, lung epithelial cells, and monocytes (Figure 4*A–D*). In line with these effects on ACE2 expression, uptake of SARS-CoV-2 S protein-expressing VLPs was elevated in IFN-γ-treated control and PTPN2-KD cells (Figure 4*D–H*). Silencing of STAT1 using small interfering RNA (siRNA) constructs prevented IFN-γ-induced ACE2 mRNA and protein expression in PTPN2-KD Caco-2BBe (Figure 5*A, B*) whereas STAT1-siRNA-treated PTPN2-KD A549 and THP-1 cells expressed ACE2 levels comparable to those in Ctr cells (Figure 5*C, D*). STAT1 silencing also reversed the elevated SARS-CoV-2 Spike protein uptake observed in PTPN2-KD cells (Figure 5*D–E*). This strongly suggests that deletion of PTPN2 promotes ACE2 expression and SARS-CoV-2 entry in a STAT1-dependent manner, and inhibition of STAT signaling may mitigate elevated ACE2 expression and SARS-CoV-2 entry into host cells.

### The Clinical PTPN2 Loss-of-function Variant, rs1893217, Increased Live SARS-CoV-2 Virus Invasion and STAT-mediated ACE2 Expression

**Figure 6:**
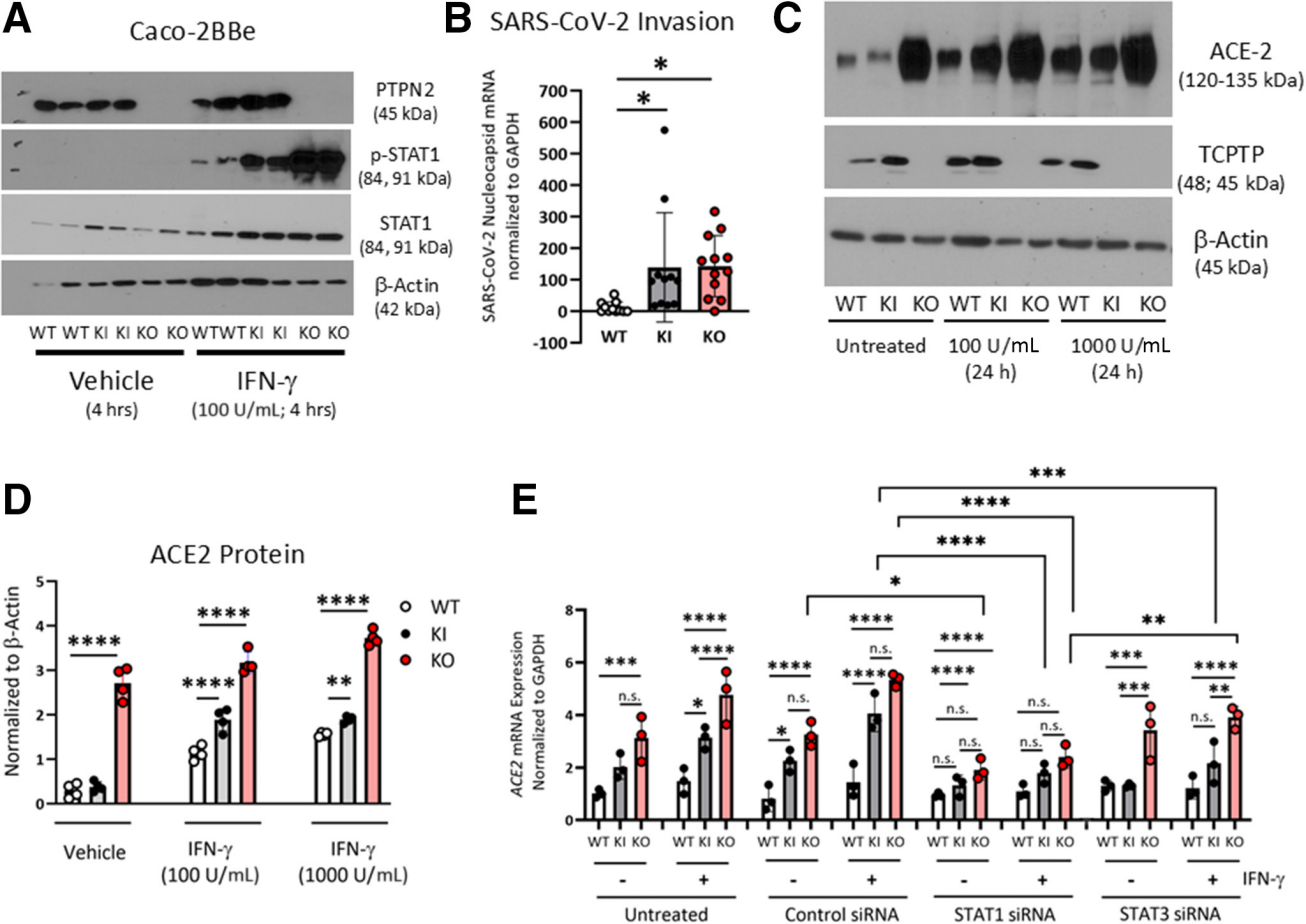
**The clinical *PTPN2* loss-of-function variant, rs1893217, increased live SARS-CoV-2 virus invasion and STAT-mediated ACE2 expression.** (*A*) Caco-2BBe cells were CRISPR-Cas9 modified to knock-in WT *PTPN2*, knock-in the *PTPN2* rs17893217 loss-of-function variant (KI) or to delete PTPN2 (KO) and subsequently challenged with vehicle (PBS) or IFN-γ (100 IU/mL) for 4 hours. Western blotting showed that KI cells expressed PTPN2, whereas KO cells had no detectable PTPN2 protein expression. PTPN2 loss-of-function was demonstrated in KI and KO cells by elevated tyrosine phosphorylation of STAT1 (Y701) following IFN-γ challenge (n = 4). (*B*) WT, KI, KO cell monolayers were incubated with live SARS-CoV-2 virus (MOI 0.7) for 1 hour analysis for SARS-Cov-2 nucleocapsid mRNA expression (n = 12). (*C*) Caco-2BBe cells were treated with IFN-γ (1000 IU/mL) for 24 hours and analyzed for ACE2 protein level (Western blot). Each dot represents the average of 1 independent experiment with 2 to 3 technical replicates. (*D*) Densitometric analysis of Western blots (n = 4). (*E*) WT, KI, and KO Caco-2BBe cells were treated with non-targeting control (siCtr), STAT1-specific (siSTAT1) siRNA, or STAT3-specific (siSTAT3) siRNA and grown as monolayers prior to incubation with IFN-γ (1000 IU/mL) for 24 hours. ACE2 mRNA expression was determined by RT-PCR. Statistical differences are indicated in the figure (1-way ANOVA, with Tukey post-test n = 3). Each dot represents the average of an independent experiment with 2 to 3 technical replicates.

To functionally interrogate the influence of the clinical *PTPN2* loss-of-function rs1893217 variant on the susceptibility to SARS-CoV-2 infection, we generated intestinal epithelial Caco-2BBe cell lines that were CRISPR/Cas9-modified to express the *PTPN2* rs17893217 variant (knock-in; KI). Confirmation of reduced PTPN2 activity was demonstrated by Western blots showing elevated phosphorylation of the PTPN2 substrate, STAT1(Y701), following IFN-γ challenge (Figure 6*A*). PTPN2-KI and KO cell lines showed increased infection with live SARS-CoV-2 virus compared with IECs carrying WT PTPN2 (Figure 6*B*). Western blot and densitometric analysis showed that PTPN2-KI and KO cells exhibited higher expression of ACE2 following challenge with IFN-γ, and at baseline in the case of PTPN2-KO cells (Figure 6*C, D*). siRNA transfection studies revealed that IFN-γ induced upregulation of ACE2 in PTPN2-KI and PTPN2-KO cells was driven by STAT1, and to a lesser extent STAT3-mediated transcription (Figure 6*E*).

### Tofacitnib Reversed ACE2 Overexpression and Increased SARS-CoV-2 Entry in PTPN2-deficient Human Cells and Mice

**Figure 7:**
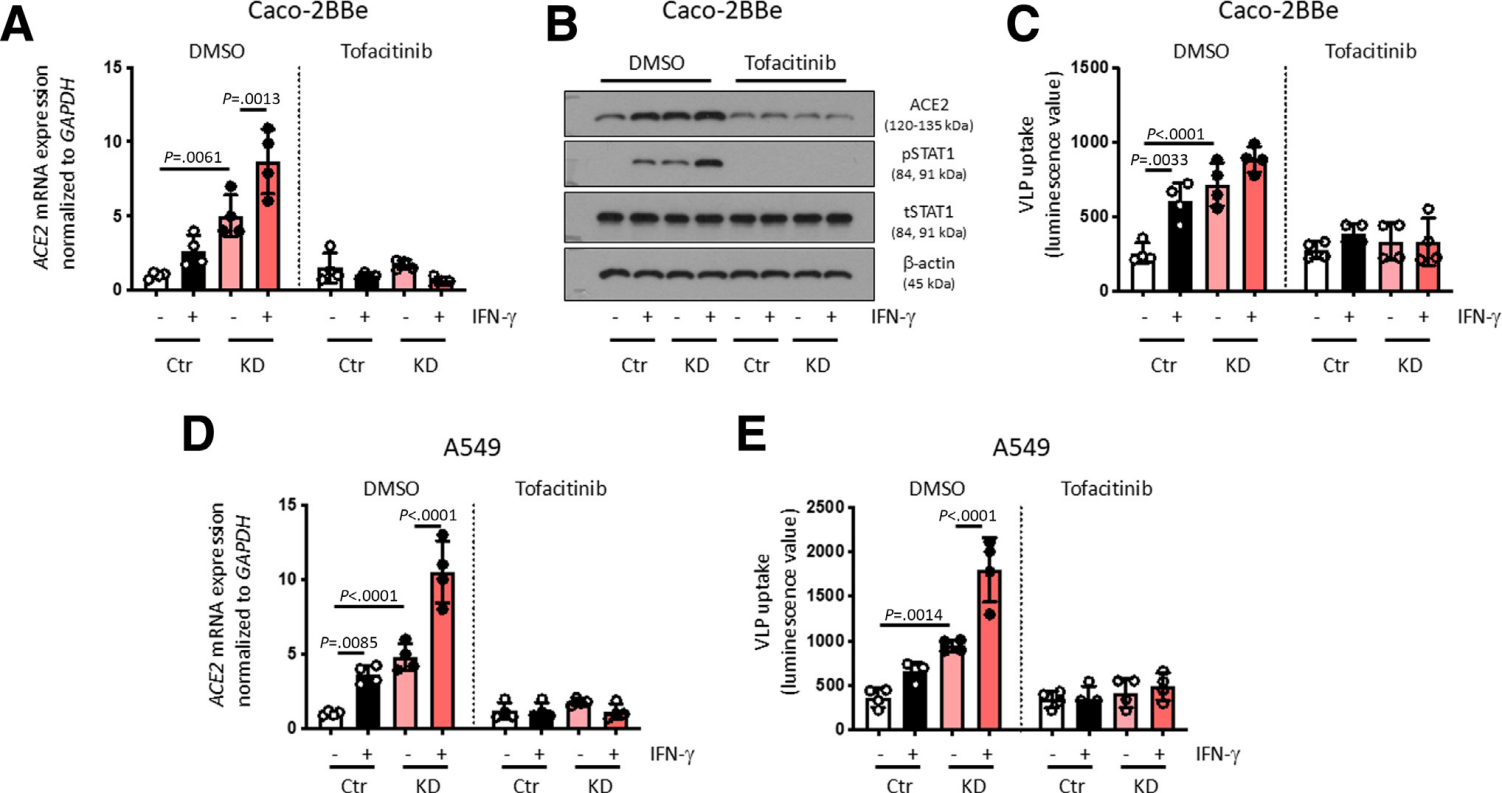
**Tofacitinib prevented epithelial ACE2 upregulation and reduced SARS-CoV-2 VLP-uptake.** (*A–C*) Caco-2BBe cells, and (*D–E*) A549 cells, expressing non-targeting control (Ctr) or *PTPN2-*specific (KD) shRNA were treated with vehicle (DMSO) or tofacitinib for 1 hour prior to infection with VLPs expressing SARS-CoV-2 spike protein in the presence or absence of IFN-γ (1000 IU/mL). (*A*) Relative mRNA expression of *ACE2* (normalized to *GAPDH*) and (*B*) representative Western blot pictures for the indicated proteins 24 hours after VLP treatment. (*C*) Luminescence values as an approximation of VLP uptake after 48 hours. (*D*) Relative *ACE2* mRNA expression in A549 cells, and **(***E*) luminescence values as an approximation of VLP uptake in A549 cells after 48 hours. Statistical differences are indicated in the figure (1-way ANOVA, *A–E*: n = 4). Each dot represents the average of an independent experiment (*A–C*) with 2 to 3 technical replicas each.

**Figure 8:**
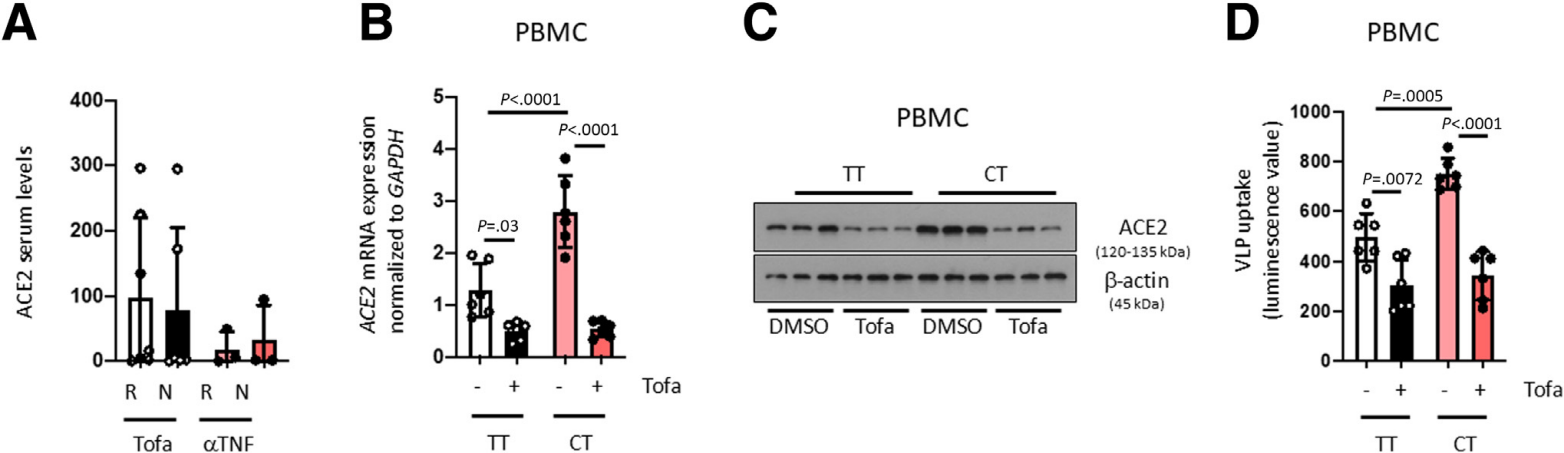
**Tofacitinib reduced ACE2 overexpression and the enhanced uptake of SARS-CoV2 spike S protein by PBMCs from IBD patients carrying the *PTPN2* rs1893217 clinical variant.** (*A*) Serum from ulcerative colitis patients treated with tofacitinib or anti-TNFα were analyzed for ACE2 protein level (R, responder; NR, non-responder). (*B–D*) Peripheral blood mononuclear cells from patients with IBD homozygous for the major allele (TT) or heterozygous for the disease-associated *PTPN2* risk allele in SNP rs1893217 were treated with vehicle (DMSO) or tofacitinab for 24 hours and analyzed for (*B*) ACE2 mRNA and (*C*) ACE2 protein expression. (*D*) After 24 hours tofacitinib-treatment, the cells were infected with VLPs expressing SARS-CoV-2 spike protein and luminescence analyzed after 48 hours as an approximation for VLP uptake. Statistical differences are indicated in the figure (1-way ANOVA, *A*, Tofacitinib-treated patients n = 12; anti-TNF-treated patients n = 6); *B–D*, n = 6). Each dot represents the average of a biological sample with 2 to 3 technical replicas each.

To test whether pharmacological inhibition of STAT activation can prevent elevated ACE2 expression in PTPN2-deficient cells, we treated Caco-2BBe IECs with the pan-JAK-inhibitor tofacitinib. Similar to our findings in cells treated with STAT1 siRNA, inhibition of JAK-STAT signaling in Caco-2BBe cells using tofacitinib prevented IFN-γ-induced increases in ACE2 mRNA and protein expression and normalized the elevated ACE2 levels in PTPN2-KD cells (Figure 7*A, B*). Moreover, tofacitinib reduced ACE2-mediated uptake of SARS-CoV-2 spike-expressing VLPs in both PTPN2-KD and in IFN-γ-treated cells (Figure 7*C*). Similar effects were observed in A549 lung epithelial cells (Figure 7*D, E*), indicating that tofacitinib treatment might not only reduce ACE2-mediated intestinal viral uptake, but also reduce viral uptake in the respiratory tract, the primary entry site of SARS-CoV-2. We next tested if tofacitinib altered ACE2 levels in human subjects. Levels of soluble ACE2, which has been suggested to reduce viral binding to host cells,^34^ were not altered in patients with UC treated with tofacitinib when compared with patients with UC under anti-tumor necrosis factor (TNF) treatment with similar disease activity (Figure 8*A*; Table 3). This suggests that tofacitinib treatment does not reduce shedding/release of ACE2 into serum. In contrast, when analysing ACE2 levels in PBMCs from patients with IBD carrying the *PTPN2* loss-of-function SNP rs1893217, we again observed that ACE2 mRNA and protein levels and SARS-CoV-2 spike-expressing VLP entry were clearly elevated in variant carriers compared with non-carriers (Figure 8*B–D*). Notably, treatment with tofacitinib not only reduced ACE2 levels and viral entry in variant carriers, but also in non-carriers (Figure 8*B–D*). These findings indicate that loss of PTPN2 or presence of the loss-of-function variant in *PTPN2* promotes ACE2 expression and subsequently facilitates uptake of SARS-CoV-2-spike-expressing VLPs, and that treatment with tofacitinib can mitigate this potential risk by reducing ACE2 cellular expression rather than affecting release of soluble ACE2. Furthermore, our data strongly indicate that treatment with tofacitinib might not only be beneficial in *PTPN2* variant carriers, but also for non-carriers.

**Table 3:** Characteristics of Patients with UC From Which Serum Samples Were Obtained

Disease	Gender (M/F)	Medication	Anti-TNF naïve	Response to anti-TNF	Tofacitinib response	Mayo score	Endoscopic remission	Clinical response
UC	M	Tofacitinib	No	No	Yes	0	Yes	Yes
UC	M	Tofacitinib	No	Yes	Yes	1	Yes	Yes
UC	M	Tofacitinib	No	No	Yes	1	Yes	Yes
UC	M	Tofacitinib	No	No	Yes	1	Yes	Yes
UC	M	Tofacitinib	No	No	Yes	0	Yes	Yes
UC	M	Tofacitinib	No	No	Yes	0	Yes	Yes
UC	M	Tofacitinib	Yes	Na	Yes	0	Yes	Yes
UC	M	Tofacitinib	No	No	Yes	1	Yes	Yes
UC	M	Tofacitinib	No	No	No	2	No	No
UC	M	Tofacitinib	No	No	No	3	No	No
UC	F	Tofacitinib	No	No	No	3	No	No
UC	M	Tofacitinib	No	No	No	3	No	No
UC	M	Tofacitinib	No	No	No	3	No	No
UC	M	Infliximab	No	Yes	No	0	Yes	Yes
UC	M	Adalimumab	No	Yes	No	0	Yes	Yes
UC	M	Infliximab	No	Yes	No	0	Yes	Yes
UC	M	Adalimumab	No	No	No	3	No	No
UC	M	Adalimumab	No	No	No	3	No	No
UC	M	Infliximab	No	Partial	No	2	No	No

F, female; M, male; TNF, tumor necrosis factor; UC, ulcerative colitis.

## Discussion

Our data consistently demonstrate that reduced PTPN2 activity, caused by gene knockdown, deletion, or expression of the clinical loss-of-function rs1893217 variant in *PTPN2*, promotes expression of the SARS-CoV-2 receptor, ACE2, and promotes the uptake of SARS-CoV-2 spike protein and live SARS-CoV-2 virus, an effect that was further increased in the presence of inflammatory stimuli. This is striking, given findings that non-genotyped patients with IBD (± inflammation) showed no change in ACE2 or TMPRSS2 expression, whereas experimental colitis in mice reduced gut epithelial *Ace2* expression.^35^ This indicates that inflammation does not promote ACE2 expression *per se,* but that the elevated ACE2 levels in patients or mice with reduced PTPN2 activity are indeed a consequence of PTPN2 deficiency and not a secondary effect.

Contrasting outcomes between studies and between disease subtypes (CD vs UC) and the innate heterogeneity of disease progression and presentation between patients render it unclear if patients with IBD have an increased risk for (severe) COVID-19, but it is clear that there is an intersection between IBD and COVID-19 related pathways.^36^ In IBD, ACE2 levels vary by disease (UC ∼unchanged; CD lower); activity (higher in active vs inactive UC) and region (ileum vs colon), whereas differential expression may also influence responsiveness to IBD therapies.^35^, ^36^, ^37^, ^38^, ^39^ In mucosal biopsies, active disease increased *ACE2* in UC—which may increase virus entry—whereas patients with CD had lower ACE2, which may increase pathogenic roles of its substrate, AngII.^35^^,^^37^ One study did identify a group of patients with CD with high *ACE2* expression in ileum and colon, and these individuals were significantly more likely to undergo surgery within 5 years of CD diagnosis. Moreover, high *ACE2* levels were concluded to be an independent risk factor for surgery.^39^ With respect to virus infection, increased ACE2 would likely increase virus uptake, but the virus subsequently reduces ACE2 expression, which could deplete anti-inflammatory functions of ACE2 and thereby worsen outcomes in COVID-19 and IBD.^37^ Thus, a complex duality of higher or lower gut ACE2 expression exists that can exert negative outcomes on the risk of initial infection vs subsequent inflammation.

Our studies also confirmed that elevated ACE2 expression—at least in the context of reduced PTPN2 activity—is mediated by STAT1/3 activity. STAT1 and STAT3 are substrates of, and are directly dephosphorylated by, PTPN2. These findings not only add to our understanding of how ACE2 is transcriptionally regulated, but also provide a mechanistic explanation for the efficacy of tofacitinib in alleviating ACE2 overexpression, and spike protein entry, into PTPN2-deficient cells.^40^ Indeed, these findings may be broadly applicable to the demonstrated efficacy of tofacitinib when co-administered with corticosteroids, in alleviating clinical symptoms and risk of death in hospitalized patients with COVID-19, including in an African-American cohort.^41^, ^42^, ^43^ Identification of genetic variants associated with SARS-CoV-2 infection and COVID-19 outcomes has been an area of intense focus. The Genetics of Mortality in Critical Care (GenOMICC) study compared genomes from critically ill individuals with those of population controls to find underlying disease mechanisms.^44^ Five of the variants associated with critical COVID-19 had direct roles in IFN signaling and shared broadly concordant predicted biological effects. These included a protein-coding variant in TYK2, a member of the JAK family that was associated with increased TYK2 expression. This was further supported in a follow-up study of 24,202 COVID-19 cases that also identified a SNP in the JAK1 locus associated with disease severity.^45^ This finding supported the rationale for clinical trials with the JAK inhibitor, baricitinib, and emphasizes the important contributions of JAK signaling in COVID-19, but also their value as therapeutic targets.^46^

Here, we demonstrate that the IBD risk SNP rs1893217 in *PTPN2* is associated with increased expression of ACE2 and SARS-CoV-2 entry and might represent a novel COVID-19 genetic susceptibility biomarker. By using samples collected well before the COVID-19 outbreak, our identification of a genetic susceptibility marker avoids the potential for ascertainment bias in most genetic studies of COVID-19, as patients with clinically significant COVID-19 are more likely to be included in research projects than asymptomatic cases.^47^ With respect to genetic markers of COVID-19 susceptibility, studies have proposed the involvement of ABO blood groups, with blood group O associated with lower risk, whereas blood group A was associated with higher risk of acquiring COVID-19 compared with non-A blood groups.^47^, ^48^, ^49^, ^50^ However, this correlation did not culminate in therapeutic implications. Moreover, a cluster of genes on chromosome 3 has been linked with increased severity, although this may have distinct geographic distributions.^47^^,^^51^ In contrast, our finding of increased ACE2 expression, along with increased viral particle/live virus uptake in *PTPN2* variant cells, might not only indicate a potentially novel genetic marker for more severe disease, but also identifies tofacitinib—a drug already approved for treatment of arthritis and IBD—and potentially other JAK inhibitors, such as baricitinib, as a potential therapeutic strategy to specifically mitigate this risk.

## Methods

### Patient Samples

Samples from patients with IBD used for RNA and protein isolation were obtained from the Swiss IBD cohort, and the sample use was approved by the local ethic’s board (Ethic’s board of the Kanton Zurich, Switzerland; approval number EK1977). Serum samples were obtained from University of California San Diego under IRB Protocol # 131487. All patients provided informed consent.

### Mice

PTPN2-KO mice in the BALB/c background were a gift from Prof M. Tremblay at McGill University in Montreal. To generate mice lacking PTPN2 specifically in intestinal epithelial cells (ΔIEC mice), mice with a loxP-flanked exon 3 of the PTPN2 gene (PTPN2-fl/fl mice, originally obtained from EUCOMM, abbreviated as fl/fl) were crossed with VillinCre-ERT2 mice (Jackson Laboratories). Translocation of the Cre-ERT2 construct and subsequent recombination and deletion of the floxed gene was induced by tamoxifen-injections (intraperitoneally, 1 mg/mouse/day) on 5 consecutive days. All mouse experiments were conducted according to protocols approved by the Institutional Animal Care and Use Committee of the University of California, Riverside (AUP20190032).

### Protein Isolation and Western Blotting

Protein isolation and Western blotting were performed according to standard procedures. For protein isolation from cells, the cells were washed with ice cold phosphate buffered saline (PBS) and lysed in radioimmunoprecipitation assay (RIPA) buffer containing phosphatase and protease inhibitors (Roche). For mouse and human biopsies, the samples were dissociated in RIPA buffer using a bead-beater and metal beads. All samples were then sonicated for 30 seconds and centrifuged (10 minutes at 12,000 g at 4 °C), and the supernatant was transferred into fresh tubes. Protein concentration was detected using a BCA assay (Thermo Fisher Scientific). For Western blots, aliquots with equal amounts of protein were separated by electrophoresis on polyacrylamide gels, and the proteins blotted on nitrocellulose membranes. The membranes were then blocked in blocking buffer (3% milk, 1% bovine serum albumin [BSA] in tris-buffered saline with 0.5% Tween) for 1 hour and incubated overnight at 4 °C with anti-ACE2 (Clone E-11, Santa Cruz Biotechnology), anti-phospho-STAT1 (Tyr701, clone 58D6; Cell Signaling Technologies), anti-STAT1 (clone 42H3; Cell Signalling Technologies), or anti-β-actin (Clone AC-74, Sigma-Aldrich) antibodies. On the next day, the membranes were washed in tris-buffered saline with 0.5% Tween, incubated with horseradish peroxidase (HRP)-coupled secondary antibodies (Jackson Immunolabs), washed again, and immunoreactive proteins detected using ELC substrate (Thermo Fisher Scientific) and X-ray films (GE Healthcare).

### PTPN2 Phosphatase Assay

For PTPN2 activity measurements, 100 μg protein lysates were pre-cleared for 1 hour using Sepharose A beads, incubated with 2 μL anti-PTPN2 antibody (Clone D7T7D, Cell Signaling Technologies) over night, incubated with Sepharose A beads for 1 hour and centrifuged (3 minute at 12,000 g at 4 °C). The precipitates were washed 3 times with ice cold PBS, the beads were resuspended in phosphatase assay buffer (Thermo Fisher Scientific), and phosphatase activity was measured using the EnzCheck Phosphatase assay (Thermo Fisher Scientific) according to the manufacturer’s instructions.

### RNA Isolation and qPCR

For RNA isolation, biopsies were disrupted in RLT buffer (Qiagen, Valencia, CA) using a bead beater and metal beads. Cells were washed twice in ice-cold PBS before lysis in RLT buffer. All samples were then passed 3 to 5 times through a 26G needle prior to RNA isolation using the RNAeasy mini kit from Qiagen. RNA concentrations were estimated by absorbance measurement at 260 and 280 nm, and cDNA generated using the qScript reverse transcriptase (Quantabio). Quantitative real-time PCR was performed using iQ SYBR Green Supermix (BioRad) on a C1000 Thermal cycler equipped with a CFX96 Real-Time PCR system using BioRad CFX Manager 3.1 Software. Measurements were performed in triplicates using *GAPDH* as an endogenous control. Results were analyzed by the ΔΔCT method. The real-time PCR included an initial enzyme activation step (3 minutes, 95 °C) followed by 45 cycles consisting of a denaturing (95 °C, 10 seconds), an annealing (53°–60°C, 10 seconds) and an extending (72 °C, 10 seconds) step. The primers used are listed in Table 4.

**Table 4:** Primers Used in the Present Study

Species	Target	Primer name	Sequence
Mouse	*Ace2*	mAce2_Fwd	3'-TGAACACCATGAGCACCATT-5'
Mouse	*Ace2*	mAce2_Rv	3'-TGCCCAGAGCCTAGAGTTGT-5'
Mouse	*Gapdh*	mGapdh_Fw	3'-CATCACTGCCACCCAGAAGACTG-5'
Mouse	*Gapdh*	mGapdh_Rv	3'-ATGCCAGTGAGCTTCCCGTTCAG-5'
Human	*ACE2*	hACE2_Fw	3'-GGACCCAGGAAATGTTCAGA-5'
Human	*ACE2*	hACE2_Rv	3'-GGCTGCAGAAAGTGACATGA-5'
Human	*TMPRSS2*	hTMPRSS2_Fw	3'-CAAGTGCTCCAACTCTGGGAT-5'
Human	*TMPRSS2*	hTMPRSS2_Rv	3'-AACACACCGATTCTCGTCCTC-5'
Human	*TMPRSS4*	hTMPRSS4_Fw	3'-CCAAGGACCGATCCACACT-5'
Human	*TMPRSS4*	hTMPRSS4_Rv	3'-GTGAAGTTGTCGAAACAGGCA-5'

### VLPs and Measurement of VLP Uptake

To produce pseudoparticles, 293T cells were transfected with plasmids encoding a minimal HIV (pTRIP, CSGW, CSPW) provirus expressing the Gaussia Luciferase (Gluc), gag-pol, and S protein of SARS-CoV-2 virus using polyethylenimine transfection reagent.^52^, ^53^, ^54^ Supernatants were collected at 24, 48, and 72 hours post-transfection, pooled, filtered (0.45 mm), aliquoted, and stored at −80 °C. Pseudoparticle infections were performed in the presence of 4 μg/mL polybrene. Appropriate amounts of pseudoparticle were added onto target cells and plates incubated for 3 hours (37 °C) before changing media. Gaussian luciferase was measured at 24, 48, and 72 hours after infection.

To measure VLP uptake into cells, the VLP-containing medium was diluted 1:2 in cell culture medium and applied to the cells. After 1 hour, the cells were washed with PBS and fresh medium added to the cells. In experiments with IFN-γ (1000 IU/mL; Roche), the replacement medium for cells infected in presence of IFN-γ contained IFN-γ as well. To determine VLP uptake, luciferase luminescence in cell culture supernatant was determined using the Renilla luciferase activity assay from Thermo Fisher Scientific.

### Live SARS-CoV-2 Virus Infection

All procedures involving live SARS-CoV-2 were conducted in a BSL-3 facility at the UCR, School of Medicine. The SARS-CoV-2 WA1 strain, sourced from Dr Rong Hai's laboratory (UCR), was propagated in Vero 6 cells, which were seeded in 75T flasks 24 hours prior to infection in Dulbecco's Modified Eagle Medium (DMEM) (high glucose) supplemented with sodium pyruvate, 4500 mg/l L-glutamine, 100× penicillin-streptomycin, and 10% fetal bovine serum (FBS). The infection was initiated with media containing 2% FBS for 24 hours, followed by a media change to regular conditions for 5 days before collection.

### Viral Plaque Assay

#### Preparation

Vero cells were plated in DMEM with 10% FBS in 12-well plates to ensure a 100% confluent monolayer by the day of the experiment.

#### Infection and incubation

Six serial 1:10 dilutions of virus samples in PBS were prepared. The diluted virus was added to the monolayer, the plate was rocked every 5 to 10 minutes for 1 hour at 37 °C to ensure even distribution and to prevent drying. The virus inoculum was then aspirated, and the cells were overlaid with 1 mL of Plaquing Media (DMEM containing 1% P/S, 1% Avicell, and 2% FBS).

#### Fixation and staining

Cells were fixed overnight with 3.7% formaldehyde in PBS, washed twice with tap water, and stained with 1 mL of 1% crystal violet for 45 minutes. Plaques were counted manually to determine virus titers.

### Cell Culture, PTPN2 KD, siRNA Treatment, and IFN-γ Treatment

HT-29.cl19A were obtained from Kim Barrett (University of California, San Diego, California). Caco-2BBe, A549 and THP-1 cells were originally obtained from ATCC and cultured according to the manufacturer’s recommendation in medium with 10% FBS. For PTPN2 KD, the cells were infected with lentiviral particles containing non-targeting control short hairpin RNA (shRNA) (Ctr) or PTPN2-specfic shRNA as described previously,^55^ and stable clones were selected using puromycin. For STAT1 silencing, the cells were transfected with previously validated, STAT1-specific or non-targeting control siRNA constructs (Dharmacon) using DharmaFECT transfection reagents as described previously.^56^ In experiments with STAT1 siRNA and IFN-γ treatment, the culture medium was replaced with serum-free medium 8 hours prior to addition of IFN-γ (1000 IU; Roche). Insertion of *PTPN2* SNP *rs1893217* (*PTPN2*-KI) and complete KO of *PTPN2* (*PTPN2*-KO) in Caco-2 BBe cell lines was performed using CRISPR-Cas9 gene editing by Synthego. In experiments with tofacitinib, the cells were treated with tofacitinib (50 μM, MedChemExpress). Control cells were treated with an equal amount of vehicle (dimethyl sulfoxide [DMSO], 0.5%, Sigma-Aldrich).

### Enzyme-linked Immunosorbent Assay

Human ACE2 enzyme-linked immunosorbent assay (ELISA) was obtained from R&D and performed according to the manufacturer’s instructions with undiluted serum.

### RNA Sequencing

RNA sequencing (RNA-seq) was performed and analyzed by the Integrative Genomics Core, City of Hope National Medical Center. The RNA-seq libraries were constructed with Kapa mRNA HyperPrep Kit (Roche) following the manufacturer’s recommendation. The libraries were then sequenced on an Illumina Hiseq 2500 with single end 50 bp reads to a depth of about 35M. The sequences were aligned to human genome assembly hg38 using Tophat2 v2.0.14. RNA-seq data quality was evaluated using RSeQC v2.5. For each sample, expression counts for Ensembl genes (v92) were summarized by HTseq v0.6.1 and reads per kilobase of transcript per million mapped reads (RPKM) were calculated. Count normalization and differential expression analyses between groups were conducted using Bioconductor package “DESeq2” v1.14.1. Heatmaps were generated using R package “heatmap3.” The GO and pathway analysis was performed using DAVID online tools and Ingenuity Pathway Analysis (IPA).

### Statistics

Data are represented as mean of a series of n biological repetitions ± standard deviation (SD). Data followed a Gaussian distribution, and variation was similar between groups for conditions analyzed together. Significant differences were determined using GraphPad Prism v9 software by analysis of variance (ANOVA). *P*-values below .05 were considered significant. Mice for ex vivo analyses were matched for age and sex. Numbers of replicates are given in the figure legends. No data points were excluded from statistical analysis.
